# Treatment of primary squamous cell carcinoma of the endometrium and review of previous literature: A case report

**DOI:** 10.1097/MD.0000000000033667

**Published:** 2023-04-28

**Authors:** Liyun Song, Qi Wu, Suning Bai, Ren Xu, Xiaona Wang, Yanyan Yang

**Affiliations:** a Department of Gynecology, Hebei General Hospital, Shijiazhuang, China.

**Keywords:** chemoradiotherapy, endometrium, high microsatellite instability, primary squamous cell carcinoma

## Abstract

**Rationale:**

Primary squamous cell carcinoma of the endometrium (PSCCE) has been occasionally reported. Treatment of this disease poses a challenge to clinicians because of its rarity. Herein, we report the case of a 56-year-old woman with typical clinical manifestations and a pathological diagnosis classified by molecular typing as having high microsatellite instability (MSI-H) PSCCE. Based on a review of the previous literature, we summarized the treatment options for this rare disease and proposed new opinions.

**Patient concerns:**

A 56-year-old woman was admitted to our hospital with irregular vaginal bleeding and lower abdominal swelling.

**Diagnosis:**

The patient was diagnosed with squamous cell carcinoma of the endometrium (stage IIIC1; MSI-H).

**Interventions:**

The patient underwent total abdominal hysterectomy, bilateral salpingo-ovariectomy (bso), and pelvic lymph node dissection. Following the surgery, the patient received adjuvant chemoradiotherapy.

**Outcomes:**

The patient was followed up regularly. No recurrence or metastasis has been reported to date.

**Lessons:**

Curettage specimens may show only well-differentiated squamous epithelium, which is indistinguishable from normal squamous epithelium. It is difficult to infer from the histological morphology that the curettage specimens originate from the uterine cavity, which makes it difficult to diagnose PSCCE before the operation. We suggest that when an imaging examination indicates a tumor in the uterine cavity, even if multiple curettage specimens indicate normal or well-differentiated squamous epithelium, it indicates the possibility of PSCCE.

## 1. Introduction

Primary squamous cell carcinoma of the endometrium (PSCCE) is an exceedingly rare entity that mostly occurs in postmenopausal women, with an average onset age of approximately 67. However, there are also cases reported in women of reproductive age and during the perimenopausal period.^[1–4]^ Since the first case of PSCCE was reported by Gebhard in 1892, only a few cases have been reported. The main clinical manifestation of PSCCE is postmenopausal bleeding, followed by vaginal discharge and pelvic pain in some patients. The urethral orifice, vaginal orifice, peritoneal surface, lungs, liver, and brain are common sites of distant metastasis.^[5]^ Fluhmann established 3 pathological criteria for PSCCE in 1928: no coexistence of PSCCE and endometrial adenocarcinoma; no connection between endometrial tumors and squamous epithelium of the cervix; and no coexistence of primary cervical squamous cell carcinoma and PSCCE.^[6]^ PSCCE is difficult to diagnose using uterine curettage alone. A significant number of patients may undergo multiple examinations and curettage before a definitive diagnosis.^[5]^ The final diagnosis mainly depends on the pathological examination after a total hysterectomy (Thyst). The gross specimen should be carefully examined to exclude the extension of the cervical tumor to the endometrium and endometrial adenosquamous carcinoma. However, the etiology and pathogenesis of PSCCE remain unclear. Owing to the scarcity of cases, there is no consensus on the treatment of this disease. Thyst with bilateral salpingo-ovariectomy (bso) was reported as the first choice treatment, especially in postmenopausal women.^[5,7,8]^ The efficacy of radiotherapy (rad) and chemotherapy (chemo) as adjunctive therapies is still uncertain.^[9]^ Here, we report a new case of PSCCE with typical clinical manifestations and a pathological diagnosis classified as high microsatellite instability (MSI-H) PSCCE by molecular typing. Based on a review of previous literature, we summarized the treatment options for this rare disease and recommended using molecular typing to guide immunotherapy for PESCC.

## 2. Case report

A 56-year-old multiparous postmenopausal Chinese woman presented to a local county hospital with a 4-month history of a small amount of vaginal bleeding and lower abdominal swelling. A gynecologic ultrasound examination showed that the patient had uterine fibroids, and observation was advised, so no relevant treatment was administered; she subsequently visited our department on 2022-08-09 for further treatment. She underwent menarche at 14 years old and menopause at 51 years old with no postmenopausal bleeding prior to her recent symptoms. She had a BMI of 23.67 and did not receive hormone supplementation following menopause.

**Figure 1.**
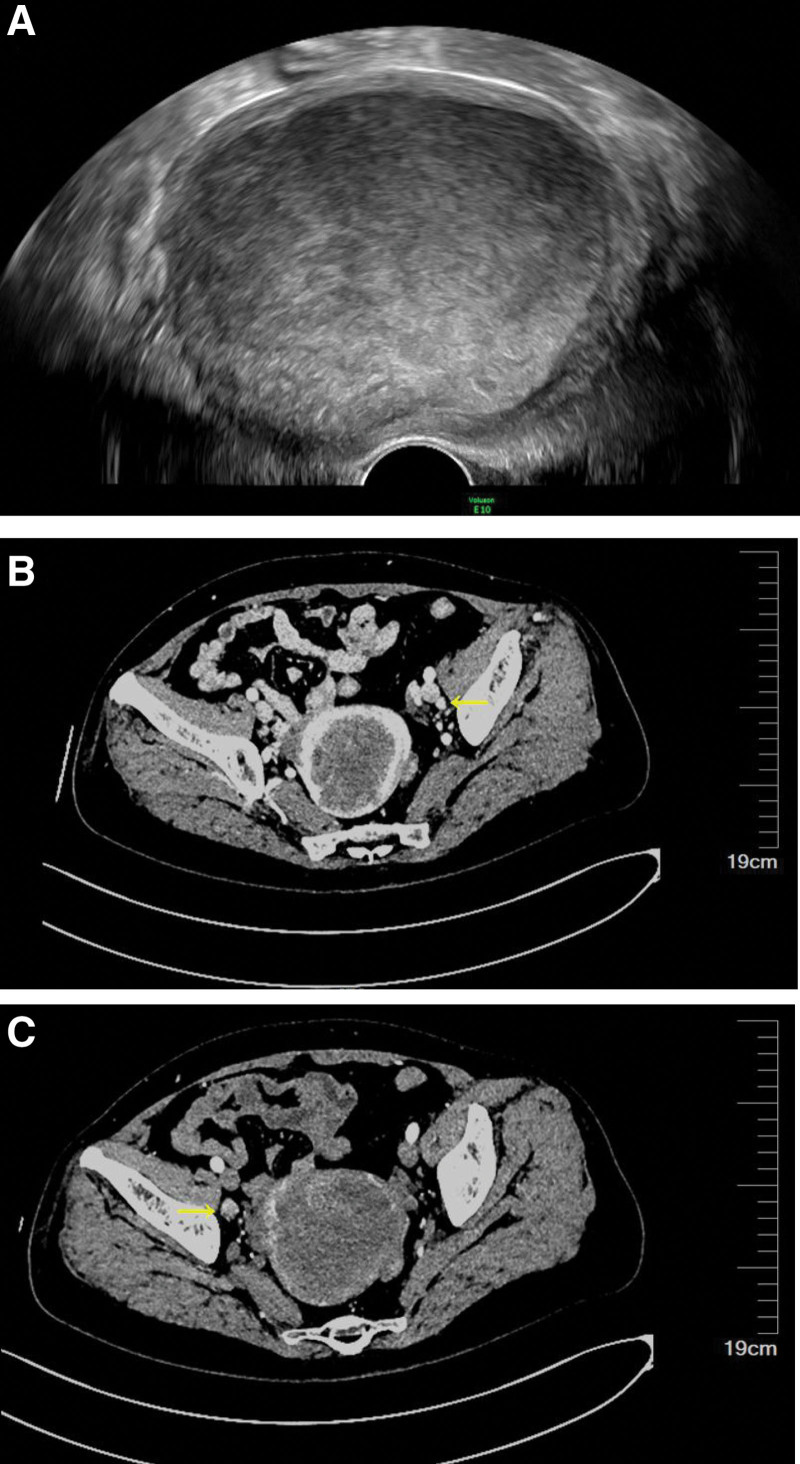
(A) Transvaginal ultrasonography revealed a hypointense, internally heterogeneous, space-occupying lesion measuring 90 × 77 × 73 mm in the uterine cavity. (B and C) Multiple lymph nodes beside bilateral iliac vessels in the pelvic cavity, some of which were swollen, were considered possible sites of metastasis.

The patient had never undergone a routine gynecological examination. The patient had a history of hypertension for >3 years, which was well-controlled. She denied a family history of malignant tumors. After hospitalization, the patient was given a physical examination, with no abnormalities in the cardiopulmonary examination and no enlargement of superficial lymph nodes. There was no tenderness or abnormal mass detected on the abdominal examination. No visible signs of abnormality on the cervix were observed during the speculum examination. On bimanual examination, the uterus was enlarged to the size of 8 weeks of pregnancy, and the patient had tenderness of the uterus and the bilateral adnexa. The human papillomavirus (HPV) and Thin Prep cytologic test of the cervix were negative. Transvaginal ultrasonography revealed a hypointense, internally heterogeneous, space-occupying lesion measuring 90 mm × 77 mm × 73 mm in the uterine cavity (Fig. 1A).

Contrast-enhanced computed tomography of the whole abdomen showed that the uterine cavity occupied space, suggesting malignant lesions. Multiple lymph nodes beside the bilateral iliac vessels in the pelvic cavity, some of which were swollen, were considered possible sites of metastasis (Fig. 1B and C). Several small lymph nodes were located behind the peritoneum. Her serum ferritin level was 227.700 ng/mL.

Based on the patient symptoms and auxiliary examination, she might have had endometrial mesenchymal sarcoma. Given the financial difficulties of the patient, it was decided not to perform a preoperative hysteroscopy to verify the diagnosis. We then proposed a diagnostic curettage or endometrial sampling, yet the patient refused since she was informed of the potential for a missed diagnosis. The patient asked for an operation, and if deemed necessary, intraoperative frozen pathology was dispatched to aid in identifying and influencing the surgical treatment strategy. Total abdominal hysterectomy combined with bso and pelvic lymph node dissection was performed on August 10, 2022. During the surgery, 200 mL of peritoneal washing fluid was reserved for cytological examination. On gross examination, the uterus was enlarged, and both adnexa appeared normal. After comprehensive exploration, no abnormal nodules were observed in the pelvic or abdominal cavities. Dissection of the uterus showed a solid mass in the uterine cavity that was brittle in texture, and gray purulent mos-like tissue could be seen in the section. Frozen pathological examination showed that a malignant uterine tumor had infiltrated the myometrium of the uterine wall.

**Figure 2.**
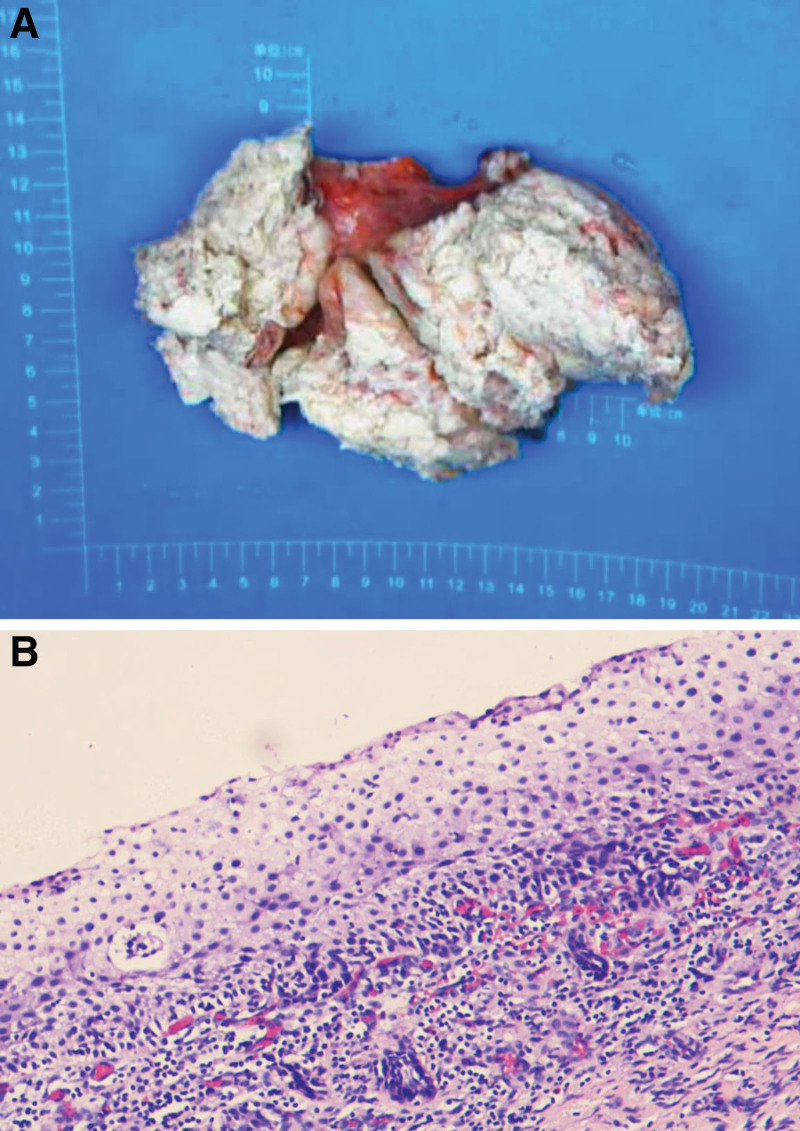
Postoperative pathological diagnosis. (A) Gross features of the uterus. (B) Histopathology of the uterus. Hematoxylin–eosin staining, 100×.

Macroscopically, the uterus measured 16 × 11 × 6 cm, and the cervix was 3 cm long with an outer diameter of 2.5 cm. The endometrial thickness was 0.2 cm, and the local endometrium was thickened. A large tumor, approximately 10 × 6 × 4 cm, was observed in the deformed uterine cavity. It was brittle, gray, and yellow in sections, and necrotic (Fig. 2A).

**Figure 3.**
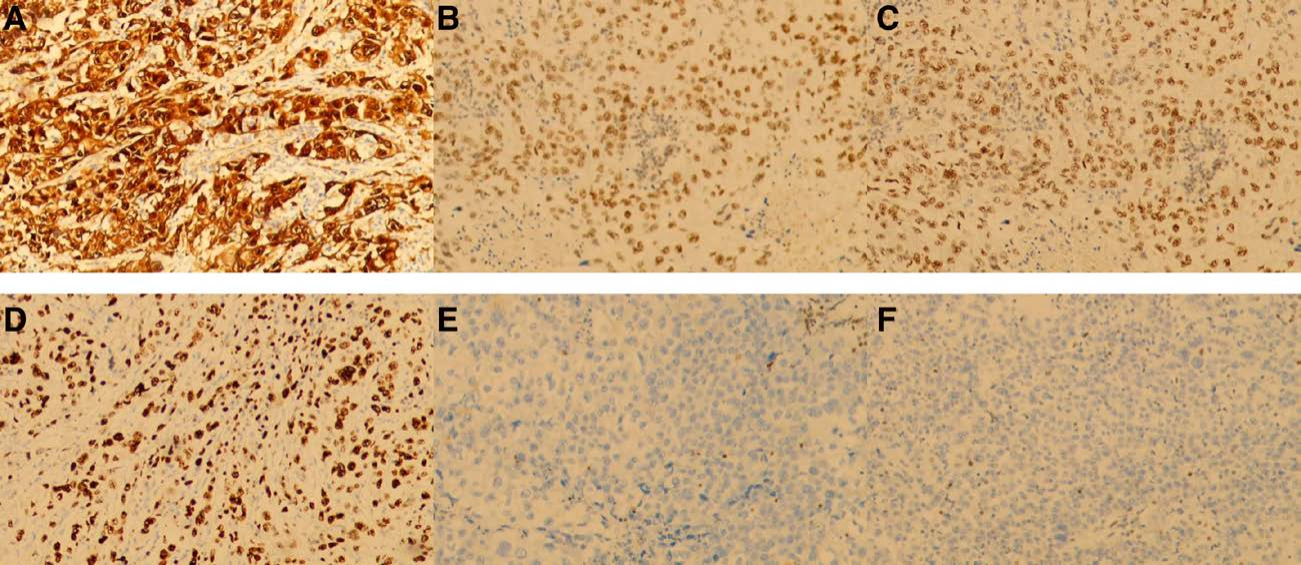
Postoperative pathological diagnosis. (A) p16 immunohistochemistry. 3,30-Diaminobenzidine staining, 100×. (B) Ki-67 immunohistochemistry (positive region 70%). 3,30-Diaminobenzidine staining, 100×. (C) MLH1 immunohistochemistry. 3,30-Diaminobenzidine staining, 100×. (D) PMS2 immunohistochemistry. 3,30-Diaminobenzidine staining, 100×. (E) MLH6 immunohistochemistry. 3,30-Diaminobenzidine staining, 100×. (F) MSH2 immunohistochemistry. 3,30-Diaminobenzidine staining, 100×.

Histochemical staining showed that poorly differentiated squamous cell carcinoma infiltrated the myometrium of the uterine wall by >1/2 (Fig. 2B). No lymphatic vascular tumor embolism was observed. No evidence of adenocarcinoma was found. No squamous metaplasia or dysplasia was observed. No cancer was found in the mucosa or stroma of the cervical canal or soft tissues around the left or right uterus. Moderate chronic inflammation was observed in the uterine cervix. During the operation, 18 pelvic lymph nodes were removed, and cancer cells metastasized to both obturator lymph nodes. No cancer was found in the peritoneal washings. Immunohistochemical analysis showed positive for Ckpan, Vimentin, CK5/6, P40, P16 (Fig. 3A), CK7, Ki-67 (positive region 70%) (Fig. 3B), MLH1 (Fig. 3C), PMS2 (Fig. 3D), weakly positive for MSH6 (Fig. 3E), and negative for SMA, Desmin, CD10, HMB45, Melan A, and MSH2 (Fig. 3F).

Histopathologically, the definitive diagnosis was squamous cell carcinoma of the endometrium (stage IIIC1, MSI-H). Ten days after the surgery, the patient received paclitaxel (albumin-bound) (260 mg/m^2^) combined with carboplatin (AUC-5) chemo for 2 to 3 days. The patient was then transferred to the oncology department for chemoradiotherapy and is alive with no evidence of recurrence or metastasis.

The patient provided written informed consent for participating in and publishing this case report.

## 3. Discussion

Most squamous cell carcinomas found in the endometrium have a primary cervical origin. Primary squamous cell carcinoma is rare, and it accounting for <1% of all malignancies of the corpus uteri.^[10]^ However, primary or secondary endometrial squamous cell carcinomas is rare, and PSCCE in situ is even rarer. The whole uterus and cervix should be sampled to meet the diagnostic criteria for PSCCE to avoid missing small lesions of endometrioid adenocarcinoma or cervical squamous cell carcinoma. Some researchers questioned Fluhmann diagnostic criteria, which classified squamous cell carcinoma involving both the endometrium and cervix as of cervical origin but failed to recognize that it might be multifocal squamous cell carcinoma occurring in the cervix and endometrium.^[10,11]^ However, the current diagnostic criteria have not changed. Here, we report a case of PSCCE that was typical of both clinical manifestations and pathological diagnoses. Curettage specimens may show only well-differentiated squamous epithelium, which is indistinguishable from normal squamous epithelium. It is difficult to infer from the histological morphology that the curettage specimens originate from the uterine cavity, which makes it difficult to diagnose PSCCE before the operation. Although most patients undergo multiple examinations and biopsies before the hysterectomy, only 50% of women are diagnosed with PSCCE before the operation.^[5]^ We suggest that when an imaging examination indicates a tumor in the uterine cavity, even if multiple curettage specimens indicate normal or well-differentiated squamous epithelium, it indicates the possibility of PSCCE.

Various controversial hypotheses have been proposed to explain the etiology and pathogenesis of PSCCE, but none have been formally accepted. Zidi et al suggested that PSCCE could arise from squamous metaplasia of the normal endometrium.^[12]^ Horn and Bilek suggested that PSCCE may be the result of the bi-directional differentiation of pluripotent endometrial precursor cells.^[13]^ Yamamoto et al suggested that PSCCE may arise from heterotrophic cervical tissue.^[14]^ Notably, Kataoka et al demonstrated the presence of HPV type 31 in a patient with PSCCE using a polymerase chain reaction.^[8]^ Horn et al performed an analysis with HPV typing in 8 patients with PSCCE and found that 1 case was HPV16 positive, and the others were negative.^[15]^ However, other reports have not shown a clear association between HPV infection and PSCCE. A study conducted by Bures et al suggested that PSCCE exhibits molecular alterations involving the pRB-cyclin D1-CDK4/6-p16 pathway and pTEN.^[9]^ Compared with type I endometrial carcinoma, PSCCE has a unique pathogenesis.^[9]^ No evidence of squamous metaplasia, atypical hyperplasia, or HPV infection was found in our patient, and no cancer was found in the mucosa or stroma of the cervical canal.

Immunohistochemical analysis plays a particular role in prompting the aggressiveness and malignancy of the tumor, clarifying its pathogenesis, and guiding postoperative adjuvant therapy. Among the reported cases of PSCCE, some were P16-negative, and most were ER and PR-negative.^[4,9,10,16,18]^ According to Hopkins et al, ER and PR expression may be limited.^[10]^ Most studies have demonstrated a mutation in the p53 tumor suppressor gene, and Ki-67 was reported with a high labeling index, indicating the aggressive and malignant nature of the lesion.^[2,4,10,16,19,20,21]^ In our patient, P16 was positive, and Ki-67 had a high labeling index (70%). Unfortunately, we did not detect ER, PR, and P53 expression. We classified this case by molecular typing: PMS2 (+), MSH2 (−), MSH6 (weakly +), and MLH1 (+), and the results confirmed that this case was MSI-H. This has a guiding role in the choice of postoperative adjuvant therapy. Owing to the rarity of PSCCE, the description of this disease is mainly based on sporadic case reports. Many patients have only short-term follow-ups, and there are few survival data after treatment. As a result, there is currently no standard treatment for this type of endometrial cancer. No single center will ever produce a series of patients of any significant size to collect a large number of complete follow-up data, perform statistical analyses, and evaluate the effectiveness of primary surgery and adjuvant therapy. We submit a new list of patients (Table 1) that fully meets Fluhmann criteria. No meaningful incidence statistics were available. Nearly all reports on PSCCE do not report the survival data well, and the follow-up period is short. The median age of the 31 patients meeting Fluhmann criteria was 64 years (range 28–90), 22 of 31 (91%) were in stage I, and 5 of 31 (16%) were dead of the disease a median of 46 months after diagnosis (11–100 months). 26 of 31 (84%) patients were alive and free of disease after a median follow-up of 14 months. 10 of the 22 patients in stage I only underwent surgery with a median follow-up of 12 months (6–180 months), while 7 received adjuvant rad after surgery with a median follow-up of 36 months (6–180 months), and more patients received rad than chemo after the operation. Postoperative adjuvant chemoradiotherapy was administered to most patients with stages III and IV disease. The primary treatment for PSCCE was a total or radical hysterectomy, followed by bso or/and lymph node resection.20 of 31 (65%) patients underwent a Thyst; 11 (35%) underwent a radical hysterectomy, and 19 (61%) underwent lymph node resection during the operation. Previous data suggest that PSCCE may have mixed behavior resembling both endometrial and cervical cancers.^[22]^ Therefore, women with early-stage cancer have a favorable prognosis; however, the survival rate is usually poor in cases of locally advanced cancer.^[10]^ According to statistics, within the survival period of 14 to 36 months, the 1-year survival rates of stage I, stage III, and stage IV of endometrial carcinoma are 80%, 20%, and 0%, respectively,^[4,5,7,22,24]^ while the prognosis of PSCCE is worse than that of endometrioid carcinoma.^[4,5,7,22,24]^

**Table 1:** Cases of primary squamous cell carcinoma of the endometrium meeting Fluhmann criteria.

Case	Ref.	Yr	Author	Age	Stage	Treatment^a^	Follow-up^b^
1	^[25]^	1993	Dalrymple et al	55	I	Thyst/rso	55 mo
2	^[25]^	1993	Dalrymple et al	64	I	Rhyst/lnd/chemo	114 mo
3	^[25]^	1993	Dalrymple et al	47	I	Rhyst/bso/lnd/rad	73 mo
4	^[24]^	1995	Kennedy et al	56	I	Thyst/bso/rad/chemo	28 mo
5	^[33]^	1999	Chung et al	68	I	Thyst/bso	6 mo
6	^[34]^	2001	Seo et al	62	I	Rhyst/bso/lnd/rad	2 mo
7	^[26]^	2001	Rodolakis et al	68	I	Thyst/bso/rad	54 mo
8	^[26]^	2001	Rodolakis et al	61	I	Rhyst/bso/lnd/rad	36 mo
9	^[35]^	2002	Varras et al	61	I	Thyst/bso	60 mo
10	^[36]^	2002	Houissa-Vuong et al	64	I	Thyst/bso/lnd/rad	DOD 46 mo
11	^[37]^	2003	Tong et al	57	I	Rhyst/bso/lnd/rad	9 mo
12	^[38]^	2008	Tchabo et al	90	I	Thyst/bso	12 mo
13	^[39]^	2011	Bifulco et al	48	I	Rhyst/bso/lnd	12 mo
14	^[21]^	2012	Lee et al	54	I	Thyst/bso/lnd/rad	13 mo
15	^[40]^	2013	Terada et al	72	I	Rhyst/bso/ome/lnd/rad/chemo	15 mo
16	^[22]^	2014	Bogani et al	74	I	Rhyst/bso/lnd	180 mo
17	^[22]^	2014	Bogani et al	67	I	Thyst/bso	DOD 100 mo
18	^[22]^	2014	Bogani et al	73	I	Thyst/bso	12 mo
19	^[41]^	2015	Jetley et al	60	I	Thyst/bso	7 mo
20	^[4]^	2019	Darre et al	28	I	Thyst/rad/chemo	3 mo
21	^[17]^	2022	Caulkins et al	65	I	Thyst/bso/lnd/chemo	24 mo
22	^[42]^	2022	Puljiz et al	62	I	Thyst/bso/lnd	12 mo
23	^[25]^	1993	Dalrymple et al	76	III	Thyst/rad	NED 21 mo
24	^[43]^	1997	Jung et al	66	III	Thyst/bso/lnd/rad/chemo	12 mo
25	^[44]^	2003	Kim et al	65	III	Rhyst/bso/lnd/chemo	12 mo
26	^[27]^	2012	Takeuchi et al	66	III	Rhyst/bso/ome/lnd/rad/chemo	22 mo
27	^[22]^	2014	Bogani et al	66	III	Thyst/bso/lnd/rad/chemo	DOD 46 mo
28	^[3]^	2018	Zhang et al	47	III	Thyst/bso/lnd/rad/chemo	DOD 11 mo
29	Song	2022	Present case	56	III	Thyst/bso/lnd/rad/chemo	5 mo
30	^[45]^	1995	Sorosky et al	72	IV	Thyst/bso/chemo	DOD17 mo
31	^[2]^	2018	Wu et al	33	IV	Rhyst/bso/lnd/ome/rad/chemo	13 mo
32	^[28]^	2020	Purbadi et al	57	IV	Thyst/bsa/rad/chemo	17 mo

a Thyst = total hysterectomy, rso = right salpingo-ovariectomy, Rhyst = radical hysterectomy, lnd = lymph node dissection, chemo = chemotherapy, rad = radiotherapy, bso = bilateral salpingo-ovariectomy, ome = omentectomy, bsa = bilateral salpingectomy.

b DOD = dead of disease, NED = no evidence of disease (alive or dead).

Goodman et al retrospectively analyzed 8 cases of PSCCE in 1995 and reviewed previous literature. They concluded that Thyst with bso is the optimal primary therapy for PSCCE.^[5]^ The prognosis was related to the clinical stage of the tumor but not to the degree of differentiation. The prognosis of stage I was good, but that of stage IV and most stage III was poor.^[5]^ The data available at that time were inadequate to evaluate the efficacy of additional rad and chemo.^[5]^

As PSCCE is rare and the available data are limited, a comparison of survival and local control between patients treated with surgery alone and surgery combined with postoperative adjuvant rad cannot be made. Thomakos and Kennedy, however, believe that adjuvant rad can treat potential micrometastases in pelvic lymph nodes to improve local and regional control.^[19,24]^ Distant metastasis indicates treatment failure; therefore, effective treatment modalities should be explored to reduce this failure. The purpose of PSCCE adjuvant chemo is to decrease distant metastasis and improve local control through radiosensitization during rad. It unclear what The curative effect of chemo is on PSCCE, but the tumor remission rate of combined chemo for cervical squamous cell tumors is 60% to 70%.^[25]^ Bogani et al suggested that adjuvant therapy with both local (rad) and systemic (platinum-based chemo) disease control should be performed for locally advanced PSCCE.^[22]^

Dalrymple et al analyzed previous cases of PSCCE with long-term survival, all of which were stage I and were treated mainly by surgery. One patient who received postoperative chemo was still alive at a follow-up of 114 months, and another patient who received postoperative rad was still alive at a follow-up of 73 months.^[25]^ A patient with stage III disease treated with surgery and adjuvant rad died 21 months after surgery, but no evidence of recurrence was found on autopsy.^[25]^ As cervical squamous cell tumors and endometrial adenocarcinomas both respond to irradiation and the characteristics of these cases are analyzed, they consider that this disease may also be radiosensitive.^[25]^ Kennedy and Caulkins reported 1 case of stage I disease, both of which were administered adjuvant chemoradiotherapy after surgery, and no evidence of recurrence was found after 28 and 24 months of follow-up, respectively.^[17,24]^ Rodolakis reported 2 cases of stage I disease, both of which were administered adjuvant rad after surgery and were followed up for 36 months and 54 months, respectively, without recurrence.^[26]^ Takeuchi reported a case of stage III disease in a patient who received adjuvant rad and chemo after surgery and was followed up for 22 months without recurrence.^[27]^ Bogani presented 2 cases in a report: a stage I case that recurred 79 months after the operation and then underwent surgery again combined with adjuvant rad, with a total survival time of 100 months, and a stage III case that recurred 21 months after the operation and received chemo, with a total survival time of 46 months.^[22]^ Purbadi and Wu reported a case of stage IV PSCCE, both of which received adjuvant rad and chemo after the operation. Chemo reduced distant metastatic lesions, and there was no recurrence of pelvic lesions during follow-up.^[2,28]^ It can be seen that postoperative adjuvant rad and chemo may prolong the overall survival of patients with PSCCE.

PSCCE is a rare subtype of endometrial cancer that is mostly described in case reports. Early diagnosis and treatment are critical because the overall prognosis is often poor. In our analysis, 19 of the 31 patients (61%) underwent lymph node resection. Lymph node metastasis is an important indicator for the pathological staging of endometrial cancer and can be used to evaluate patient prognosis. Moreover, considering that PSCCE is a special type of endometrial cancer, lymph node resection is recommended in patients with this disease. Reported cases typically receive adjuvant platinum-based chemo and/or radiation with varying efficacies.^[22,24,27]^ Based on the above considerations, our patient underwent a Thyst combined with bso and pelvic lymph node dissection and received postoperative chemo with paclitaxel and carboplatin, followed by further adjuvant chemoradiotherapy. The patient was followed-up regularly, and no recurrence or metastasis has been identified to date.

Currently, immunotherapy is being rapidly developed. Microsatellite instability (MSI) is a clonal change in the number of repeated DNA nucleotide units in a microsatellite. It arises as a result of defective mismatch repair (MMR) caused by the failure of 1 of the 4 main MMR genes: MSH2, MLH1, MSH6, or PMS2. Failure of MMR results in somatic inactivation, leading to MSI and genomic instability, which in turn leads to neoplasia.^[29]^ A study by Eggink et al showed that PD-1 was overexpressed in tumor-infiltrating lymphocytes and peritumoral lymphocytes of MSI tumors, suggesting that MSI tumors may be excellent candidates for immunotherapies targeting the PD-1 pathway.^[30]^ A non-randomized, multi-center trial by Marabelle A et al showed that the total effective rate of pabolizumab in 49 patients with endometrial cancer was 57%.^[31]^ Pembrolizumab was approved in the United States by the US Food and Drug Administration in May 2017 for the treatment of MSI-H solid tumors. Combined immunotherapy may further improve the poor prognosis of PESCC; however, its efficacy requires further investigation.

PSCCE mostly occurs in elderly postmenopausal women, and owing to the vulnerability of the elderly, a careful balance between different treatment approaches is essential.^[22,32]^ Therefore, the surgical plan should be individualized for elderly patients. Comprehensive treatment of the elderly should be performed effectively, not only to extend their life span but also to maintain the dignity of life and the expectation of health.^[22,23]^

## 4. Conclusion

Herein, we report the case of a 56-year-old woman with typical clinical manifestations and a pathological diagnosis who was classified by molecular typing as having MSI-H PSCCE. Based on a review of the previous literature, we summarized the treatment options for this rare disease and proposed new opinions. We recommend that surgery should remain the primary treatment for PSCCE, and lymph node excision should be advised. For stage I disease, adjuvant rad should be administered; however, for stages III and IV, adjuvant chemoradiotherapy should be administered. However, multi-institutional efforts are still needed to evaluate the effects of different rad and chemo regimens. Combined immunotherapy may further improve the poor prognosis of PESCC; however, its efficacy requires further investigation.

## Author contributions

**Formal analysis:** Liyun Song, Suning Bai, Xiaona Wang, Yanyan Yang.

**Investigation:** Liyun Song, Ren Xu.

**Resources:** Liyun Song.

**Software:** Liyun Song.

**Supervision:** Yanyan Yang.

**Writing – original draft:** Liyun Song, Qi Wu.

**Writing – review & editing:** Liyun Song, Qi Wu, Suning Bai, Ren Xu, Xiaona Wang.
